# Preparation of Chitosan/Magnetic Porous Biochar as Support for Cellulase Immobilization by Using Glutaraldehyde

**DOI:** 10.3390/polym12112672

**Published:** 2020-11-12

**Authors:** Haodao Mo, Jianhui Qiu

**Affiliations:** Department of Machine Intelligence and Systems Engineering, Faculty of Systems Science and Technology, Akita Prefectural University, Yurihonjo 015-0055, Japan; D18S003@akita-pu.ac.jp

**Keywords:** enzyme immobilization, covalent bonding, porous biochar, chitosan, magnetic composites

## Abstract

In this work, porous biochar was obtained from sugarcane bagasse by alkali activation and pyrolysis and then magnetized with γ-Fe_2_O_3_ by calcination. After functionalization with chitosan and activation with glutaraldehyde, the as-prepared chitosan/magnetic porous biochar served as a support to immobilize cellulase by covalent bonds. The immobilization amount of cellulase was 80.5 mg cellulase/g support at pH 5 and 25 °C for 12 h of immobilization. To determine the enzymatic properties, 1% carboxymethyl cellulose sodium (CMC) (dissolved in 0.1 M buffer) was considered as a substrate for hydrolysis at different pH values (3–7) and temperatures (30–70 °C) for 30 min. The results showed that the optimum pH and temperature of the free and immobilized cellulase did not change, which were pH 4 and 60 °C, respectively. The immobilized cellulase had a relatively high activity recovery of 73.0%. However, it also exhibited a higher Michaelis–Menten constant (K_m_) value and a slower maximum reaction velocity (V_max_) value compared to the free enzyme. In the reusability assay, the immobilized cellulase showed initial glucose productivity of 330.9 mg glucose/g CMC and remained at 86.0% after 10 uses. In conclusion, the chitosan/magnetic porous biochar has great potential applications as a support for enzyme immobilization.

## 1. Introduction

In recent years, using biomass for bioethanol production has garnered great interest. Cellulose and hemicellulose can be hydrolyzed to reducing sugars, and then the sugars can be fermented into ethanol. For the process of hydrolyzing lignocellulosic materials, the use of strong acids or alkalis increases the burden on the environment and equipment, but enzymatic hydrolysis would not. Therefore, enzymatic hydrolysis of lignocellulosic should be a greenway to produce fermentable reducing sugars [1,2,3]. Cellulase, a composite enzyme, is mainly composed of endo-l, 4-β-d-glucanase, exo-l, 4-β-d-glucanase, and β-glucosidase. Its classification is based on attacking the depolymerization stage of the substrate. Endoglucanases randomly hydrolyze the glycosidic bonds in the amorphous regions of cellulose to produce oligomers with several degrees of polymerization. Then, exoglucanase hydrolyzes the β-1,4-glycosidic bond of the oligomer to produce cellobiose. Finally, cellobiose is degraded to glucose by β-glucosidase [4]. However, some factors limit the application of free cellulases, such as changes in pH, temperature, and ionic strength, product inhibition, and difficulty in recovering from the reaction medium. Therefore, it is meaningful to improve the stability and reusability of cellulase [5,6]. Several methods can be used for enhancing the stability of the enzyme, such as protein engineering, chemical modification, and immobilization [7,8]. Among them, immobilization has more advantages in heterogeneous enzymatic reactions and reusability [9].

Immobilization on a solid support can improve an enzyme’s stability and makes it easier to recover said enzyme from the medium and soluble substrate, as previously proven [10,11]. For a solid substrate, immobilization can also provide a way to improve the stability and reusability of enzymes. Covalent bonding can provide a stable structure for an enzyme, and an immobilized enzyme with a magnetic base can be easily separated from the respective slurry by a magnetic bar [12]. In recent years, various types of solid supports have been used for enzyme immobilization, such as natural clays [13,14], gels [15,16], and porous materials. Usually, natural materials have good biocompatibility and rich functional groups, but the low surface area limits their application. Therefore, porous materials have a large specific surface area that could provide more space for enzyme immobilization. Mesoporous silica [17], metal–organic framework materials [18], and zeolites [19] are popular supports in enzyme immobilization. However, their preparations are complicated and normally require precision, which increases the costs.

Biochar, a solid porous particle obtained by the pyrolysis of biomass in the absence of oxygen [20], is popular in soil amendments [21], wastewater treatments [22], and electrode materials [23]. Simultaneously, it is emerging as a promising support for immobilizing enzymes. Porous biochar has a high specific surface area (~1600 m^2^/g) and different types of pore structures [24,25]. Compared to other materials (e.g., mesoporous silica, zeolite, graphene, and metal–organic framework), porous biochar has the advantages of abundant sources, a simple preparation method, and a cheap cost. However, the insufficient number of reactive and hydrophilic groups and inconvenient recovery limit its application to the immobilization of hydrophilic enzymes.

In order to conveniently and quickly recycle and reuse enzymes, magnetic base material has been paid much attention because it can be easily separated from the reaction system by simply applying a magnet. [26,27,28,29]. Co-precipitation and hydrothermal methods are commonly used to prepare magnetic base materials. However, it is difficult to use these two methods to prepare magnetic biochar because there are few functional groups on the surface of activated porous biochar. If the iron ions are firstly dispersed and attached to the biochar, the magnetic particles can be uniformly grown in the biochar after calcination. This is a good strategy to prepare magnetic biochar. In order to improve the biocompatibility of magnetic biochar, chitosan is usually used to modify the supports because it has good hydrophilic, biocompatible, and non-toxicity properties. In addition, chitosan can allow amino groups to covalently bind with enzymes [30,31,32].

Covalent attachment is a very convenient method for enzymatic immobilization. It has been proven to be more efficient and can provide more stable biocatalysis [33]. Glutaraldehyde should be the most widely used cross-linking agent because it is facile, efficient, and can improve the stability of an enzyme by multipoint or multisubunit immobilization. A support with primary amino groups can be activated by glutaraldehyde; then, the glutaraldehyde-activated support reacts with the primary amino groups of the enzyme. For immobilized cellulase, the glutaraldehyde-activated carrier may be sterically hindered because of its spacer arms. However, it can be considered a hetero-functional support that can provide chemical reaction groups and anion exchange, and it offers the highest reactivity with the amino groups of a protein [34]. Epoxy- and di-vinyl-sulfone (DVS)-activated supports are also popular for enzyme immobilization via multipoint covalent attachment, but they have some limitations; for example, the low reactivity of epoxy-activated supports [34] and the low activity recovery of DVS-activated supports [35]. In addition, dithiocarbamate (DTC) is of great interest as a new functional group for covalent immobilization. The amination carrier is firstly modified with carbon disulfide to generate a DTC group, and is then covalently bonded to the amine group on the surface of an enzyme [36,37]. Though this is an effective technique and DTC has a shorter spacer arm, carbon disulfide is an enzyme inhibitor and this method still needs further discussion. Therefore, glutaraldehyde was selected as the covalent agent for cellulase immobilization in this work.

Based on the above, the main objective of this work was to use porous biochar (obtained from agricultural waste sugarcane bagasse) as a basis to obtain chitosan/magnetic porous biochar after magnetization and functionalization. Then, it was used as a support for cellulase immobilization via glutaraldehyde. The structure and morphology of the support were characterized, and the enzymatic properties of the free and immobilized enzymes were evaluated in hydrolyzed carboxymethyl cellulose sodium, including optimum pH and temperature, kinetic parameters, and reusability.

## 2. Materials and Methods

### 2.1. Materials

Sugarcane bagasse was produced in Guangxi, China. Potassium hydroxide (KOH), hydrochloric acid (HCl; 35–37 wt%), ferric chloride hexahydrate (FeCl_3_·6H_2_O), ferrous chloride tetrahydrate (FeCl_2_·4H_2_O), chitosan (CS), acetic acid (HAc), sodium acetate (NaAc), glutaraldehyde (GA; 25%, *v*/*v*), and carboxymethyl cellulose sodium (CMC) were purchased from Nacalai Tesque, Inc. (Tokyo, Japan). Cellulase (pale yellow powder) was bought from Meiji Seika Pharma Co., Ltd. (Tokyo, Japan).

### 2.2. Support Preparation

First of all, the porous biochar was prepared from the sugarcane bagasse by pyrolysis with KOH activation [38]. After boiled processing at 95 °C for 8 h, impurities on the surface of the sugarcane bagasse were removed. The dry pretreated sugarcane bagasse was mixed with KOH and ethanol at a ratio of 1 g/1 g/12 mL. The mixture was thoroughly mixed (500 r/min, 60 °C for 5 h) and then dried (60 °C for 12 h). Then, it was pyrolyzed in a tube furnace with nitrogen protection at 800 °C for 2 h (heating rate: 10 °C/min). After grinding and soaking in a 1.5 M HCl solution to remove ash and alkali, the porous biochar was washed with distilled water and dried (80 °C for 24 h), and then denoted as C.

In order to improve the combination of the porous biochar and the magnetic base, a calcination method was used [39]. First, 0.1 g of porous biochar, 0.2 mmol of FeCl_3_·6H_2_O, and 0.1 mmol of FeCl_2_·4H_2_O were dispersed in 2 mL of an ethanol solution. The mixture was calcined in a tube furnace at 500 °C for 1 h under nitrogen protection (heating rate: 10 °C/min). Finally, magnetic porous biochar was obtained and denoted as C/γ-Fe_2_O_3_.

Before cellulase immobilization, it is very effective and convenient to modify biochar with chitosan to improve its biocompatibility and to increase its surface functional groups. First, 0.5 g of C/γ-Fe_2_O_3_ was added to 25 mL of a 1% (*v*/*v*) acetic acid solution (containing 50 mg of chitosan) with strong stirring at room temperature for 30 min; then, it was mixed with 25 mL of a 1 M NaOH solution. The products were recovered by a magnet and washed with distilled water 5 times, before being denoted as C/γ-Fe_2_O_3_@CS.

### 2.3. Cellulase Immobilization

Here, C/γ-Fe_2_O_3_@CS was activated by glutaraldehyde. The support obtained above was dispersed in 25 mL of a 2.5% (*v*/*v*, dissolved in distilled water, pH 7) glutaraldehyde solution at room temperature for 2.5 h. Afterward, the activated support was washed with distilled water and a 0.1 M HAc–NaAc buffer solution (pH 5) 3 times.

In the cellulase immobilization process, the activated support was put into 25 mL of a 4 mg mL^−1^ cellulase solution (400 mg of cellulase powder was dissolved in 100 mL of a 0.1 M pH 5 HAc–NaAc buffer solution at room temperature) with low stirring at room temperature for 12 h. The products were washed with a 0.1 M pH 5 HAc–NaAc buffer solution 3 times and recovered by a magnet. The immobilized cellulase was stored at 4 °C, and the supernatant was used to determine the concentration of residual cellulase by the Bradford protein assay method [40]. The cellulase immobilization amount and yield were calculated by the following equation:

Cellulase immobilization amount = C_0_V_0_ − C_1_V_1_

where C_0_, C_1_, V_0_, and V_1_ refer to the concentration and volume before and after cellulase immobilization, respectively.

### 2.4. Characterizations

X-ray diffraction (XRD) was used to determine the structure and composition of the samples, while a scanning electron microscope (SEM; Hitachi S-4300, Tokyo, Japan) was used to analyze the morphologies of the particles. The magnetism of the samples was characterized by a vibrating-sample magnetometer (VSM; Riken Denshi Co. Ltd., Tokyo, Japan). Brunauer–Emmett–Teller (BET; Micromeritics, Norcross, GA, USA) analysis determined the average pore size, the BET surface area, and the total pore volume of the samples using the nitrogen adsorption method at 77 K. The chemical structures of the samples were confirmed by Fourier transform infrared spectroscopy (FT-IR; IRT-7000, Jasco, Tokyo, Japan). The amount of cellulase and reducing sugar were determined using a UV spectrophotometer (UV-vis; U-5100, Tokyo, Japan).

### 2.5. Activity Assay

The enzyme activity was determined using the IUPAC method [41]. The steps of the activity assay were as follows: For the free cellulase, 0.5 mL of cellulase solution (0.02 mg mL^−1^, dissolved in a 0.1 M HAc–NaAc buffer) was reacted with 0.5 mL of 1% (*m*/*v*) CMC for 30 min; for the immobilized cellulase, the samples (containing 0.2 mg of cellulase) were dispersed in 10 mL of a 0.1 M buffer solution and mixed with 10 mL of a 1% (*m*/*v*) CMC solution (both of them were preheated) for 30 min. The supernatant was used for measuring the amount of reducing sugars via the dinitrosalicylic acid (DNS) colorimetric method. The cellulase activity (IU/mg cellulase) was defined as the production of μ mol of glucose per minute through the hydrolysis of CMC by cellulase. To evaluate the effects of pH and temperature on cellulase activity, the hydrolysis reactions were carried out at different pH (3.0–7.0 at 50 °C) and temperatures (30–70 °C at pH 4).

### 2.6. Kinetic Assay

This assay was performed by measuring the glucose produced by the cellulase hydrolyzation of different concentrations of substrates at the optimum pH and temperature for 5 min. The samples (containing 0.2 mg of cellulase) were dispersed in 10 mL of a 0.1 M pH 4 HAc–NaAc buffer solution and mixed with 10 mL of different concentrations of a CMC solution (5, 7.5, 10, 12.5, and 15 g L^−1^ of a 0.1 M pH 4 HAc–NaAc buffer solution) at 60 °C for 5 min. The Michaelis–Menten constant (K_m_) and the maximum reaction velocity (V_max_) were determined by Lineweaver–Burk plots.

### 2.7. Reusability Assay

Based on the viewpoint of practical applications, a longer hydrolysis time was used to determine the reusability of the immobilized cellulase [5]. The immobilized cellulase containing 3 mg of cellulase was mixed with 10 mL of a 1% (*m*/*v*) CMC solution (pH 4) for 24 h at 60 °C. Then, the immobilized cellulase was recycled by a magnet and added to a fresh CMC solution for another cycle. The reusability was evaluated by the production of reducing sugars from each cycle, and the reusability assay was repeated 10 times.

## 3. Results

### 3.1. Characterization of Supports and Cellulases

**Figure 1:**
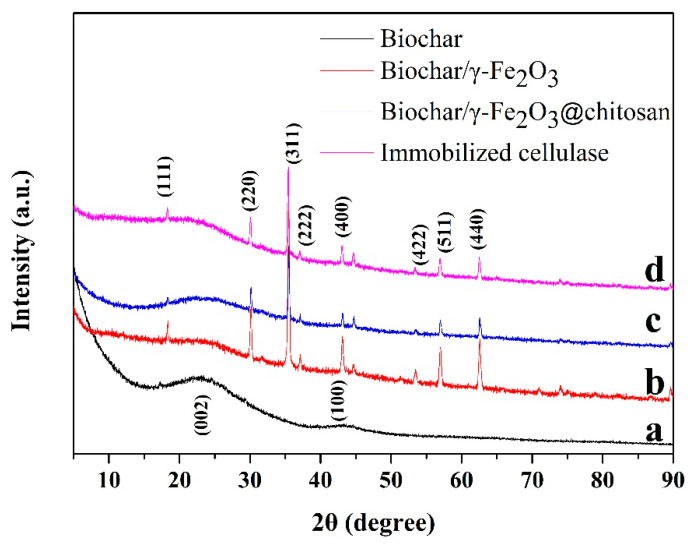
The X-ray diffraction (XRD) patterns of biochar (**a**, black curve), biochar/γ-Fe_2_O_3_ (**b**, red curve), biochar/γ-Fe_2_O_3_@chitosan (**c**, blue curve), and immobilized cellulase (**d**, pink curve).

Figure 1 shows the XRD patterns of the biochar (black curve), biochar/γ-Fe_2_O_3_ (red curve), biochar/γ-Fe_2_O_3_@chitosan (blue curve), and immobilized cellulase (pink curve). There are two broad diffraction peaks that appear at 23.1° and 43.4° in the pattern of Figure 1a, which should be the (002) and (100) planes of graphite [42]. Based on the patterns of the biochar/γ-Fe_2_O_3_, all of the diffraction peaks can be indexed to γ-Fe_2_O_3_ according to JCPDS no. 39-1346 [43], which indicates that γ-Fe_2_O_3_ grew on the surface of the porous biochar. After coating chitosan and immobilizing cellulase, the crystal of γ-Fe_2_O_3_ did not change, as indicated by the blue and purple curves.

**Figure 2:**
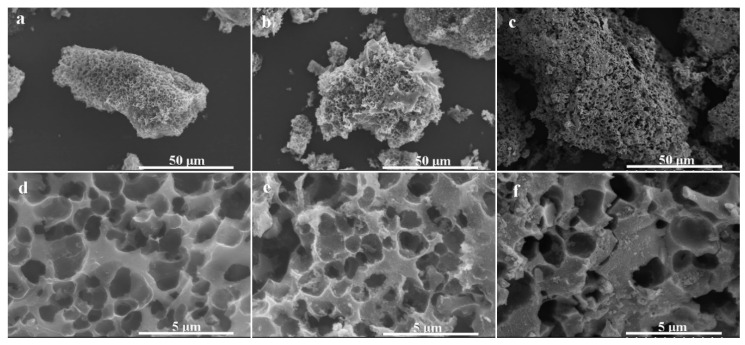
Scanning electron microscopy (SEM) images of (**a**,**d**) biochar, (**b**,**e**) biochar/γ-Fe_2_O_3_, and (**c**,**f**) biochar/γ-Fe_2_O_3_@chitosan. (**d**–**f**) Magnified images at the center of the materials in (**a**–**c**), respectively.

The SEM images of the porous biochar and its magnetic composites are given in Figure 2. A porous structure can be found in porous biochar (Figure 2d), which has a smooth surface. After mixing with the iron and calcination, a lot of crystals grew on the surface of the porous biochar, indicating that the porous biochar can be well combined with magnetic particles by calcination. As per Figure 2c,f, a thin layer can be seen covering the surface of the biochar and magnetic particles following chitosan modification, and the macropore structure of the porous biochar was well maintained, which was helpful for the substrate to diffuse into the pores so as to improve the enzymatic performance.

**Figure 3:**
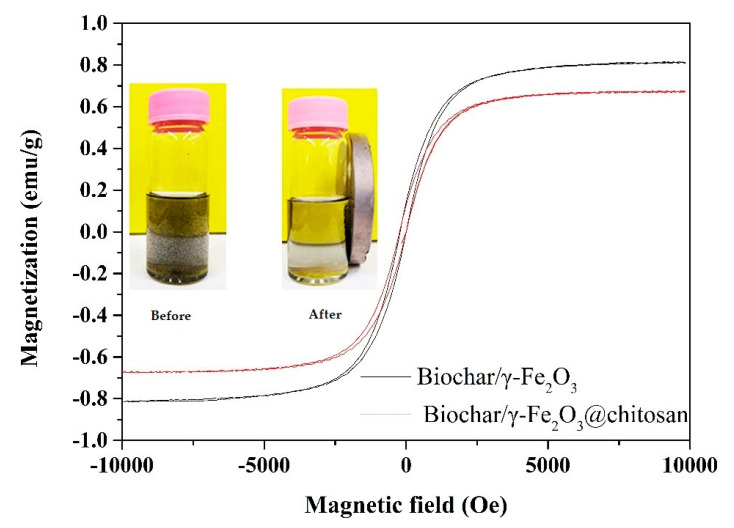
Vibrating-sample magnetometer (VSM) magnetization curves of the biochar/γ-Fe_2_O_3_ (black curve) and biochar/γ-Fe_2_O_3_@chitosan (red curve). The insets show the state of the biochar/γ-Fe_2_O_3_@chitosan before and after being recovered by a magnet.

Figure 3 shows the magnetization curves of the biochar/γ-Fe_2_O_3_ and biochar/γ-Fe_2_O_3_@chitosan at room temperature. It can be seen that the saturation magnetization of the biochar/γ-Fe_2_O_3_ was 0.81 emu/g. However, the saturation magnetization of the biochar/γ-Fe_2_O_3_@chitosan decreased to 0.67 emu/g. This could have been caused by the coating of non-magnetic chitosan on the surface of the biochar/γ-Fe_2_O_3_ and the weight conversion of the coating. It can be clearly seen from the photos in Figure 3 that most of the biochar/γ-Fe_2_O_3_@chitosan could be easily recovered by a magnet.

**Figure 4:**
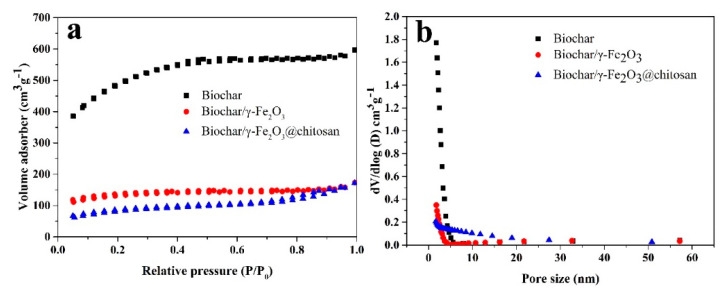
(**a**) The N_2_ adsorption–desorption isotherm and the (**b**) pore size distribution of the porous biochar (black square), biochar/γ-Fe_2_O_3_ (red ball), and biochar/γ-Fe_2_O_3_@chitosan (blue triangle) using the BJH method.

Figure 4 shows the N_2_ adsorption–desorption isotherm (Figure 4a) and the pore size distribution (Figure 4b) of the porous biochar, biochar/γ-Fe_2_O_3_, and biochar/γ-Fe_2_O_3_@chitosan using the BJH method. The nitrogen adsorption–desorption isotherms of the samples exhibit a combination of type I and IV shapes according to the IUPAC classification, which explains that the samples contain both micropores and mesopores [39]. However, the N_2_ adsorption capacity of the biochar decreased after modification with γ-Fe_2_O_3_ and chitosan. The average pore size, BET surface area, and total pore volume of the porous biochar, biochar/γ-Fe_2_O_3_, and biochar/γ-Fe_2_O_3_@chitosan are given in Table 1. After modification of the magnetic base material and chitosan, the BET surface area of the support reduced from 1595.7 to 271.6 m^2^ g^−1^. This is because, during the process of modifying the support, the modification coated the surface of the porous biochar and the inner wall of the pores, thereby blocking a part of the mesopores of the biochar. After modification, the average pore size of the sample increased. This is because the iron ions adhered to the biochar and then the magnetic iron oxide crystals grew after calcination. During this process, some of the small pores were blocked, so the average pore size of the biochar/γ-Fe_2_O_3_ became larger than that of the biochar. After chitosan modification, flocculent chitosan covered the surface of the sample, causing some of the small pores to become blocked, leading to an increase in the average pore size.

**Figure 5:**
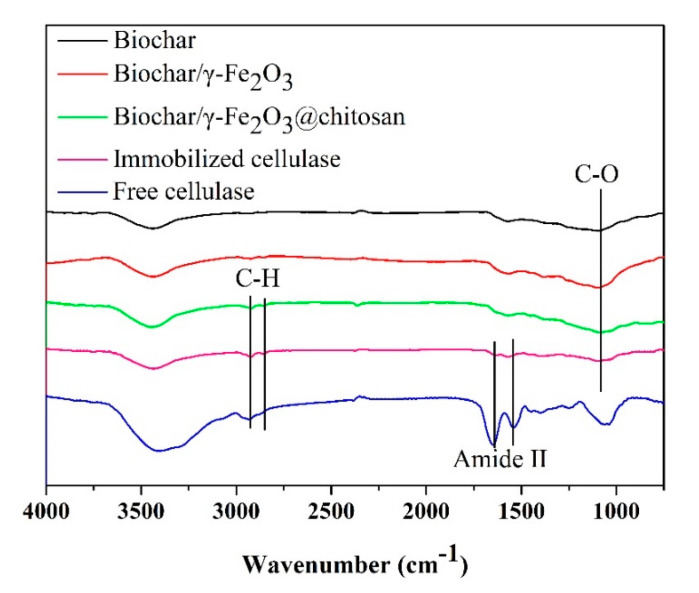
Fourier transform infrared spectroscopy (FT-IR) spectra of the biochar (**black curve**), biochar/γ-Fe_2_O_3_ (**red curve**), biochar/γ-Fe_2_O_3_@chitosan (**green curve**), immobilized cellulase (**pink curve**), and free cellulase (**blue curve**).

The chemical functional group of the biochar (black curve), biochar/γ-Fe_2_O_3_ (red curve), biochar/γ-Fe_2_O_3_@chitosan (green curve), immobilized cellulase (pink curve), and free cellulase (blue curve) samples was determined by FT-IR. As shown in Figure 5, the C–O stretching vibration of the porous biochar was found at 1100 cm^−1^ [44]. For the biochar/γ-Fe_2_O_3_@chitosan, the methylene stretching vibrations at 2924 and 2856 cm^−1^ were attributed to the chitosan layer [45]. Moreover, amide II stretching vibrations of the cellulase at 1648 and 1560 cm^−1^ were found in the immobilized enzyme, which suggests that the cellulase was successfully immobilized onto the support.

### 3.2. Effect of pH and Temperature on Cellulase Activity

According to the Bradford protein assay method and equation calculations, the amount and rate of cellulase immobilization was 80.5 mg cellulase/g support and 40.25%, respectively. In this method, multi-point or multi-subunit immobilization is such a slow process that the cellulase could be adsorbed onto the glutaraldehyde-activated support before the end. After three washings with buffer, the adsorbed cellulase was desorbed from the support. Moreover, the acidic condition was not ideal for multi-point immobilization [34]; however, the cellulase was stable under this condition.

**Figure 6:**
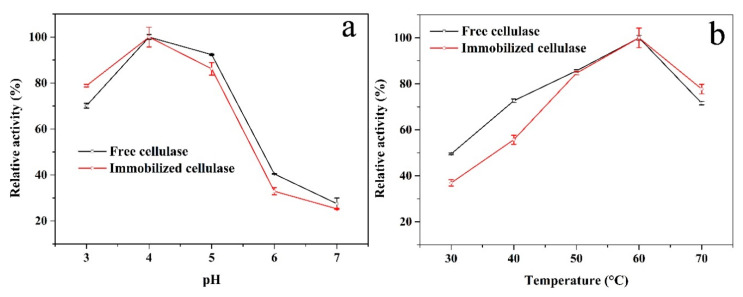
Influences of (**a**) pH and (**b**) temperature on the relative activity of the free (black curve) and immobilized (red curve) cellulase.

After immobilization, the structure of cellulase may be altered, which would change the accessibility of the active site, stability, and specificity [46]. Therefore, it is necessary to investigate the influence of pH and temperature on the activity between free and immobilized enzymes. Figure 6a shows that the relative activity of the free and immobilized cellulase had similar trends and that the optimal pH was 4. This could suggest that there are few alterations of the cellulase after immobilization. In addition, the immobilized cellulase showed higher relative activity than the free cellulase at pH 3. Under acidic conditions, the protonation of chitosan was easy and it was possible to interact with more CMC [45]. This means that during the hydrolysis process, the CMC concentration around the immobilized enzyme could have been higher than that of the free one at a lower pH, which could have promoted the hydrolysis process. The influence of the relative activity on the thermal characteristics of both enzymes was determined to be in the range of 30–70 °C at pH 4. The results are shown in Figure 6b and the highest activity appeared at 60 °C. At 30 and 40 °C, the relative activity of the immobilized cellulase was slightly lower than that of the free enzyme. The enzyme, after being covalently immobilized (compared to the free enzyme), was fixed and could not freely contact/react with the substrate in the medium. Therefore, the substrate should diffuse into the support to reach the active site of the immobilized enzyme. Moreover, the product should diffuse away from the active site to facilitate further binding of the substrate [47,48]. The diffusion coefficient is a function of temperature, increases as temperature increases. When the temperature was low, the diffusion coefficient of CMC was weak; therefore, the immobilized cellulase showed lower relative activity. However, at a high temperature, the immobilized cellulase showed higher stability. This could be because a covalent bond between the support and the enzyme protects the conformation of cellulase during heating.

**Figure 7:**
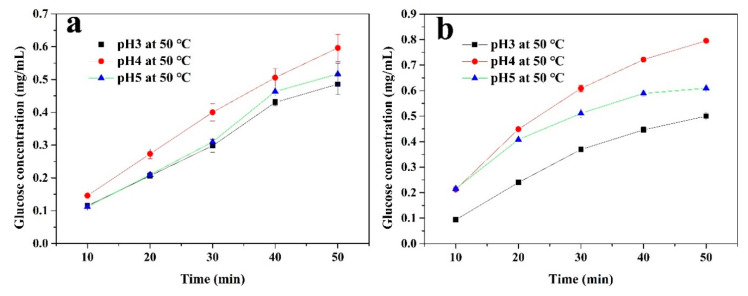
The glucose produced from the (**a**) immobilized and (**b**) free cellulase by hydrolyzing a 1% carboxymethyl cellulose sodium (CMC) solution at 50 °C with different pH values for 50 min.

**Figure 8:**
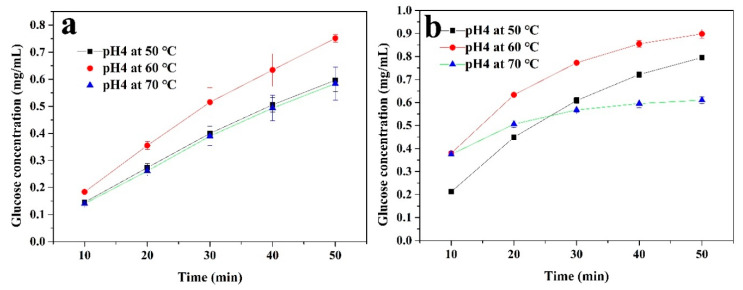
The glucose produced from the (**a**) immobilized and (**b**) free cellulase by hydrolyzing a 1% CMC solution at pH 4 with different temperatures for 50 min.

In order to further investigate the factors affecting the cellulase activity, the immobilized and free enzymes were used to hydrolyze a 1% CMC solution for 50 min at 50 °C with different pH levels (3, 4, and 5), as well as at pH4 with different temperatures (50, 60, and 70 °C). The concentration of glucose in the supernatant was measured every 10 min. As shown in Figure 7, both the immobilized and free cellulase had the highest concentrations of glucose at pH 4. The glucose produced by the free enzyme hydrolysis of CMC was higher than that of the immobilized enzyme. However, the change in pH had a greater impact on the free enzyme’s hydrolysis process of CMC. Moreover, the inhibitory effect on enzyme activity was more obvious at pH 3. The immobilized enzyme showed a relatively stable state under these conditions, which could be attributed to the covalent bonds and the CS layer, confirming the above analysis. Figure 8 shows the effect of temperature on the hydrolysis process. As the temperature increased (from 50 to 70 °C), the diffusion coefficient of CMC became stronger and it was easier for the CMC to diffuse into the active sites of the enzyme. For the free enzyme (Figure 8b), the concentration of glucose showed a higher level at 60 and 70 °C during the initial hydrolysis. However, this enzyme may denature and its original structure could be destroyed at an excessively high temperature, which would inhibit the enzyme activity. This is why the growth rate of the glucose concentration quickly returned to being flat at 70 °C. In Figure 8a, compared to the free enzyme, the glucose amount of the immobilized cellulase increased steadily during the 50-min continuous reaction. This is because this enzyme is less susceptible to temperature-induced conformational changes after covalent immobilization [49], meaning that cellulase can maintain its structure during the hydrolysis process.

### 3.3. Effect of CMC Concentrations on Cellulase Activity

**Figure 9:**
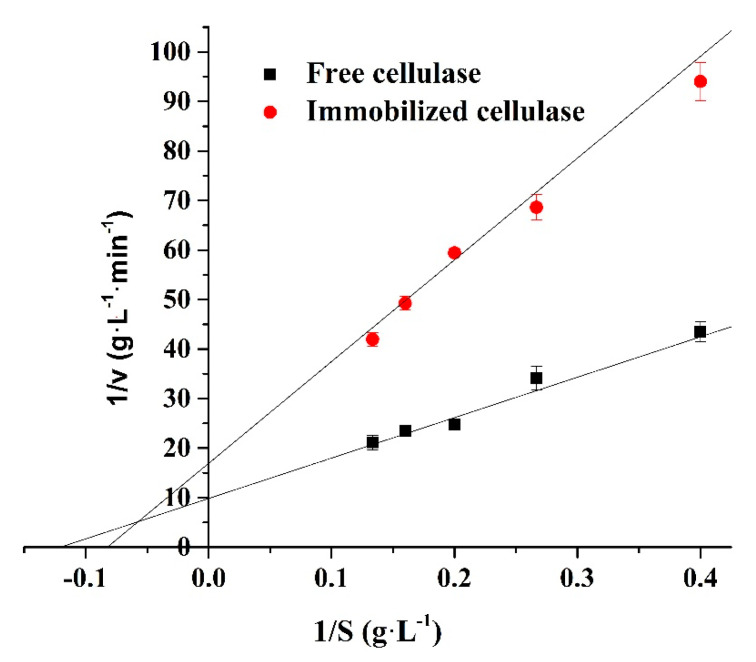
Effect of the CMC concentration on the cellulase activity (the cellulase concentration was 0.01 mg/mL at pH 4 and 60 °C for 5 min). 1/V is the reciprocal of the enzymatic reaction rate and 1/S is the reciprocal of the substrate concentration.

The Michaelis–Menten constant (K_m_) and maximum reaction velocity (V_max_) of enzymes are important kinetic parameters for determining the tightness of a substrate and enzyme binding and the speed of the enzymatic reaction. The K_m_ and V_max_ were determined by Lineweaver–Burk plots shown in Figure 9 and Table 2. The K_m_ value of the free and immobilized cellulase was 8.298 and 12.134 g L^−1^, while their V_max_ values were 0.102 and 0.059 g L^−1^ min^−1^, respectively. The increase in K_m_ and the decrease in V_max_ indicates that the binding tightness between the immobilized enzyme and substrate decreased compared to that of the free enzyme. This is because of the steric hindrance generated by the support [45].

### 3.4. Reusability of the the Immobilized Cellulase

**Figure 10:**
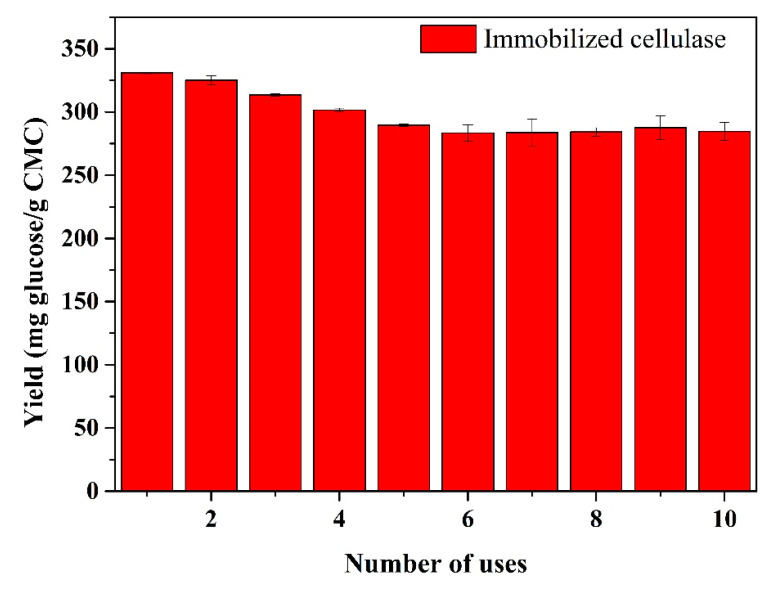
Effect of recycling on the glucose productivity of immobilized cellulase. Each cycle was performed at pH 4 and 60 °C for 24 h.

The reusability of immobilized enzymes is one of the key factors for lowering the cost in practical applications, which was evaluated herein by measuring the glucose yield in each hydrolysis cycle (Figure 10). As the number of uses increased, the glucose yield showed a slow decline and then became stable, whereas the initial glucose yield was 330.9 mg glucose/g CMC and remained at 86.0% after 10 repeated uses. The loss of glucose yield may have been caused by the protein denaturation or cellulase leakage during the hydrolysis process [5]. Moreover, it is possible that a large number of reaction products were deposited on the surface of the substrate, thereby restricting the activity of the proteins. Here, the results suggest that immobilized enzymes can be used several times with a high glucose yield, indicating their potential in practical applications.

In addition, the immobilized cellulase was stored in a refrigerator at 4 °C for one month. Then, the activity was tested. The results showed that the relative activity of the immobilized cellulase was 90.36% of the initial value, indicating its good storage stability.

## 4. Conclusions

A chitosan/magnetic porous biochar support was successfully prepared by simple methods. Cellulase was immobilized onto the support by covalent bonding using the GA agent. Under the influence of pH and temperature, the relative activity trend of the immobilized enzyme was similar to that of the free enzyme. It seems that there were few alterations of the cellulase, and the optimum temperature and pH of both the immobilized and free enzymes were 60 °C and pH 4. However, the immobilized cellulase showed high reusability: 86.0% of initial glucose productivity remained after 10 cycles. Therefore, the chitosan/magnetic porous biochar support has potential in practical applications based on the enzymatic performance of its immobilized cellulase, but it still needs further discussion.

## Figures and Tables

**Table 1:** The average pore size, Brunauer–Emmett–Teller (BET) surface area, and total pore volume of the biochar, biochar/γ-Fe_2_O_3_, and biochar/γ-Fe_2_O_3_@chitosan.

Samples	Average Pore Size (nm)	BET Surface Area (m^2^ g^−1^)	Total Pore Volume (cm^3^ g^−1^)
Biochar	2.6	1595.7	0.923
Biochar/γ-Fe_2_O_3_	3.6	421.3	0.119
Biochar/γ-Fe_2_O_3_@chitosan	3.8	271.6	0.208

**Table 2:** Kinetic parameters of the free and immobilized cellulase.

	Km (g L^−1^)	Vmax (g L^−1^ min^−1^)
Free cellulase	8.298	0.102
Immobilized cellulase	12.134	0.059
